# Endothelial Senescence in Neurological Diseases

**DOI:** 10.14336/AD.2023.0226-1

**Published:** 2023-12-01

**Authors:** Xuechun Xiao, Huimin Jiang, Huimin Wei, Yifan Zhou, Xunming Ji, Chen Zhou

**Affiliations:** ^1^Beijing Advanced Innovation Center for Big Data-Based Precision Medicine, School of Biological Science and Medical Engineering, Beihang University, Beijing, China.; ^2^Beijing Institute of Brain Disorders, Laboratory of Brain Disorders, Ministry of Science and Technology, Collaborative Innovation Center for Brain Disorders, Beijing Advanced Innovation Center for Big Data-Based Precision Medicine, Capital Medical University, Beijing, China

**Keywords:** endothelial cell, cellular senescence, blood-brain barrier, neurological disorders, anti-senescence

## Abstract

Endothelial cells, which are highly dynamic cells essential to the vascular network, play an indispensable role in maintaining the normal function of the body. Several lines of evidence indicate that the phenotype associated with senescent endothelial cells causes or promotes some neurological disorders. In this review, we first discuss the phenotypic changes associated with endothelial cell senescence; subsequently, we provide an overview of the molecular mechanisms of endothelial cell senescence and its relationship with neurological disorders. For refractory neurological diseases such as stroke and atherosclerosis, we intend to provide some valid clues and new directions for clinical treatment options.

## 1. Introduction

Cellular senescence is a physiological or pathological process that occurs throughout life, and is the predetermined fate of all cells, including endothelial cells (ECs). This state is marked by irreversible growth arrest and impairment of cell function [1]. Senescence occurs mainly in the G0/G1 phase of the cell cycle and is an important mechanism that prevents the transmission of damaged DNA to daughter cells or the potential tumor transformation of damaged cells [2]. Accumulation of senescent ECs causes the loss of cellular replicative capacity, apoptosis, and associated cardiovascular diseases [3].

ECs from highly dynamic and differentiated monolayers arranged in a vascular network. Even within brain tissue, the ECs of arteries, capillaries, and veins present different molecular characteristics [4]. The main functions of ECs as a major cellular component of the blood-brain barrier (BBB) are to express cell membrane transport proteins [5], produce of inflammatory mediators, deliver nutrients to brain tissue, and prevent drugs and toxins from entering the central nervous system (CNS) [6]. ECs are the first echelons of cells affected at the onset of senescence due to their special structural position in the vascular network. ECs undergo dysfunction from the onset of senescence, mainly in the form of a decrease in endothelium-associated vasodilators such as NO and an increase in endothelium-associated contractile factors such as endothelin [7]. Senescence ECs also produce reactive oxygen species (ROS), which directly inhibit smooth muscle potassium channels and cause vasoconstriction [8]. In addition, the vascular endothelium is in a constant process of damage and repair, and once damage occurs, ECs replenish themselves to remove the damaged cells. However, ECs senescence makes the endothelium less capable of self-repair [9]. With the decline in endothelial function, excess accumulated senescent cells express senescence-associated secretory phenotypes (SASPs), which result in the senescence of adjacent cells and eventually the degeneration of vascular function [10].

In this review, we list in detail the recent research advances in the field of EC senescence. First, we explore the phenotypes and molecular mechanisms of senescent ECs. Second, owing to the special status of ECs in the cerebral vessels, we also discuss the contribution of senescent ECs to the progression of neurological disorders and the feasibility of targeting senescent ECs for precision therapy.

## 2. The phenotype of senescent ECs

**Figure 1.**
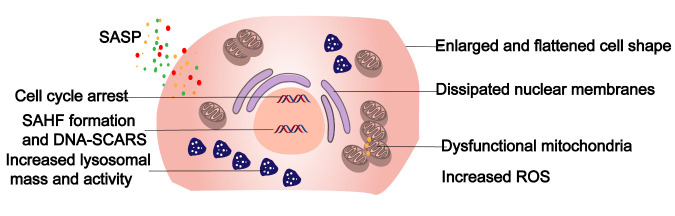
Phenotype of senescent endothelial cells. The most prominent hallmark of cellular senescence is cell cycle arrest, which occurs alongside changes in cellular phenotype, the onset of mitochondrial dysfunction, elevated ROS production, lysosomal vacuolization, elevated SA-β-Gal, and the secretion of cytokines and chemokines by senescent cells; these features are collectively referred to as senescence-associated secretory phenotypes (SASPs). In addition, dense structures (SAHF) are formed by chromatin rearrangement in senescent cells, and DNA damage accumulates. SASP, senescence-associated secretory phenotype; SAHF, senescence-associated heterochromatin foci; ROS, reactive oxygen species.

Compared with normal ECs, senescent ECs undergo a variety of pathological changes, including some highly apparent changes in cellular phenotype, as well as cell cycle arrest, the onset of SASPs, macromolecular damage, and metabolic disorders [11]. The details are shown in Figure 1.

### 2.1 Morphological changes occurring in senescence ECs

ECs, as polarized cells, are demarcated distinguished from the surrounding tissues by a glycoprotein-based membrane (secreted by ECs) on one side and by direct contact with blood on the other side, forming a functional barrier. ECs are aligned along the vessel wall, adapting to the shear stresses applied by the blood in different places. However, they are thin, presenting a flattened shape with a length of approximately 30-50 μm, width of 10-30 μm, and a thickness of approximately 0.1-10 μm. ECs cultured in vitro, usually present a typical cobblestone pattern [9, 12]. Senescent ECs become larger and flatter due to the accumulation of cellular contents associated with division in the cytoplasm and nucleus as a result of cell cycle arrest [13]. It has been suggested that cofilin1 is associated with changes in cell morphology [14]. The nuclei of senescent ECs also undergo changes, such as increased nucleus size, multinucleation, and dissipation of the nuclear membrane [15, 16].

### 2.2 Cell cycle arrest occurring in senescent ECs

The most notable feature of cellular senescence is the irreversible arrest of the cell cycle, which is one of the most fundamental indicators for identifying cellular senescence, this form of cell cycle arrest differs from quiescence and terminal differentiation [17]. ECs undergo the same senescence process as other types of cells, that is, they enter cell cycle arrest, in which the retinoblastoma (Rb) family and p53 are essentials [18]. In senescent ECs, the CDK2 repressor p21^CIP1^ (CDKN1A) and the CDK4/6 repressor p16^INK4A^ accumulate, which contribute to the sustained activation of Rb family proteins, inhibition of E2F activation in trans, and consequent cell cycle arrest, which cannot be reversed over time by subsequent inactivation of Rb family proteins or p53 [19]. Cell-cycle-related factors, such as Rb and p53, are thought to be markers of senescent cells, including ECs. The detection of p16 and p21, which are currently accepted indicators for detecting cellular senescence, permits us to understand whether ECs are in a state of senescence.

### 2.3 SASPs occurring in senescent ECs

Similar to other types of senescent cells, senescent ECs also secrete multiple factors, including proinflammatory cytokines, chemokines, angiogenic factors, and matrix metalloproteinases, thus displaying what are collectively referred to as senescence-associated secretory phenotypes (SASPs) [2]. IL-6 [20], IL-8 [21], IL-17 [22], MMP [23], and other molecules secreted by senescent ECs are considered biomarkers of cellular senescence. In senescent ECs, NF-κB and CCAAT enhancer-binding protein β, the main transcription factors involved in SASP gene regulation, are activated and enriched in chromatin fractions and regulate SASPs by directly controlling the transcription of the related molecules. Meanwhile, SASPs enhance the activity of CCAAT enhancer binding protein β and NF-κB in an autocrine feedforward loop that amplifies signaling [24, 25], SASPs also alter the phenotype of non-senescent neighboring cells through paracrine effects, thereby promoting atherosclerosis [26]. The investigation of SASP-targeted anti-senescence drugs should not be limited to effects on senescence attention should also be paid to their antagonistic pleiotropy, which is a potential side effect that may surpass their therapeutic effect [27]. Since SASP components are also associated with phenomena other than senescence, its role in physiological functions such as immune regulation is also essential. Considering all these factors, a combination of drugs may be effective in reducing potential side effects. Xu et al. revealed that cocktail therapy combining dasatinib and quercetin reduced SASP factor levels and the abundance of senescent cells in patients [28].

### 2.4 Macromolecular damage occurring in senescent ECs

The first molecular manifestation of cellular senescence is telomere shortening [29], which degrade the structural stability of telomeric DNA loops, inhibiting telomere structural damage, activating the DNA damage response, and ultimately resulting in cell cycle arrest[30]. Minamino et al. demonstrated the important role of telomeres and telomerase in vascular EC senescence, and inhibition of telomere function effectively induced the onset of senescence in human aortic endothelial cells (HAECs) [8]. Thus, telomere shortening serves as a significant biomarker of EC senescence.

Protein toxicity is another significant marker of cellular senescence [31]. ROS are a crucial source of protein damage and oxidize methionine and cysteine residues [32], which also act as indicators for the detection of senescent ECs. Yang et al. found that ROS promote vascular EC senescence via the p53 pathway [33] and Zhu et al. detected a significant elevation of ROS in senescent human umbilical vein endothelial cells (HUVECs) [34]. The amino acid residues carbonylated by ROS react with amino acids to produce Schiff bases, which promote protein aggregation and cross-linking with sugars and lipids to form lipofuscin, thereby changing protein folding and function. Lipofuscin is detected by histochemical methods using a biotin-labeled Sudan Black B analog (GL13) [35], which is also a commonly used marker to detect senescent cells.

### 2.5 Metabolic disorders occurring in senescent ECs

The increasing number of dysfunctional lysosomes in senescent cells is balanced by the production of more functional lysosomes [36]. Ren et al. invented a novel sulfur dioxide probe that regulates EC senescence, translocating to the lysosomes and protecting the V-ATPase proton channels thereon [37]. In addition, senescence-associated β-galactosidase (SA-β-gal) accumulates in lysosomes [1], and when the β-gal substrate X-gal is provided to the cells, senescent cells activate the substrate and turn it dark blue, allowing the senescent cells to be observed by ordinary functional microscopy. Detection of SA-β-gal activity by Liu [38] and Le [39] et al. confirmed the presence of senescent ECs. Although SA-β-gal is evident in senescent cells, there is an argument that it is neither necessary nor a determinant of the senescence phenotype [1].

There is an opinion that cellular senescence is best explained by a "network" theory, in which it is the inevitable consequence of multiple factors [40]. Therefore, it is difficult to determine the number of senescent cells using a single marker. In ECs, there are no specific markers for identifying endothelial senescence. Therefore, some investigators recommend using multiple markers to accurately identify senescent ECs [17]. In general, senescent ECs is detected by a combination of multiple markers: (1) markers to detect the activity of SA-β-gal or mitochondria-related alterations; (2) markers to detect the expression of p16 and p21; (3) markers to detect factors that are altered in the specific context of senescence, such as SASP and DDR molecules.

## 3. Molecular mechanisms of EC senescence

Understanding the mechanisms of EC senescence can help us explore its deeper links with senescence-related neurological diseases. Targeting these senescent ECs could be a promising treatment for some related neurological diseases.

### 3.1 EC senescence induced by cell cycle arrest

**Figure 2.**
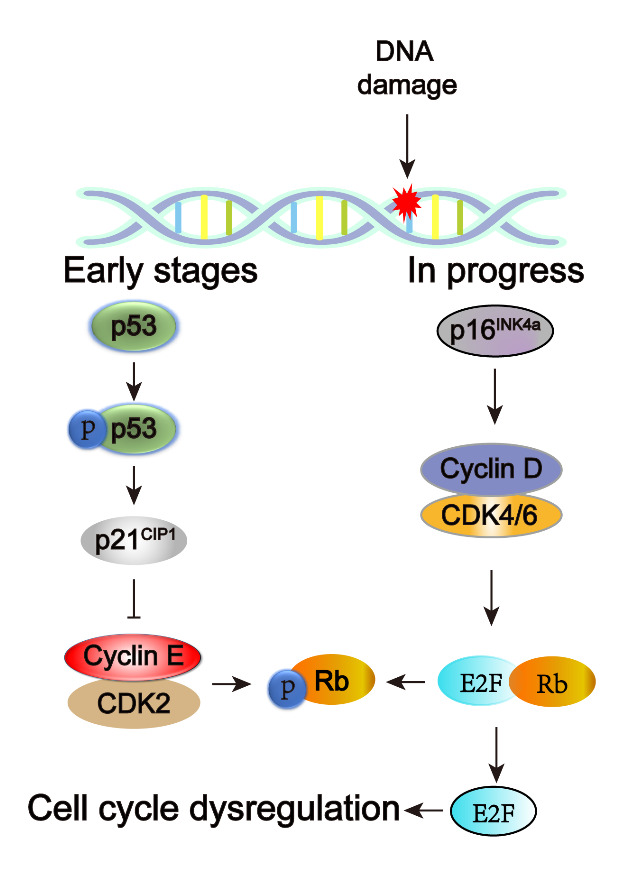
Schematic diagram of the regulation and dysregulation of the senescent cell cycle in senescence ECs. The main pathway controlling early senescence is the p53-p21 signaling pathway, whereas the maintenance of the senescent state is controlled mainly by the p16-pRb signaling pathway.

EC senescence is regulated by the p53 and p16/pRb pathways. Chen et al. showed significant upregulation of p53, p21^CIP1^ and p16^INK4a^ expression in a senescence model induced by enzymatic glycosylation of type I collagen in HUVECs [41]. Yang et al. demonstrated that UNC5B facilitates the incidence of senescence through activation of the p53 signaling pathway in vascular ECs [33]. Zhang et al. also observed that IL-17A promotes senescence by activating the NF-κB/p53/Rb signaling pathway and increasing the expression of p53, p21 and p16 in mouse aortic endothelial cells [42]. On this basis, we speculate that the occurrence of cell cycle dysregulation in ECs is similar to its occurrence in other cell types. p53 is activated by phosphorylation, which transiently upregulates the expression of p21^CIP1^. p21^CIP1^ inhibits the CDK2-CyclinE complex, thus dephosphorylating Rb and consequently dissociating E2F, resulting in dephosphorylates the Rb-E2F complex, ultimately inhibiting the cell cycle. The details are shown in Figure 2.

In the context of injury and stress, senescence and apoptosis are distinct cell fates. Proapoptotic cellular changes usually have a positive anti-senescence effect [43]. The specific inhibitor of BCL-2, BCL-XL, and BCL-W, Navitoclax (ABT-263) induces apoptosis in a variety of senescent HUVECs caused by ionizing radiation, oncogene expression, or replicative failure [44]. Second-generation inhibitors of the BCL-2 protein family, A1331852 and A1155463, have the same DNA damage-induced anti-senescence effects [45]. Chinese medicine has been used in China for more than 2,000 years, and there is no shortage of herbal monomers that exert anti-senescence effect. Zhu et al. observed that fisetin shifted senescent HUVECs toward apoptosis, but had no killing effect on non-senescent cells [46]. Zheng demonstrated that ginsenosides Rb1 activated SIRT1 and induced AMPK phosphorylation, which upregulated NOS expression levels and inhibited PAI-1 expression, thereby protecting HUVECs from H_2_O_2_-induced senescence [47]. Similarly, Shi et al. demonstrated that Ginsenoside Rb1 counteracted LDL-induced senescence in HUVECs via the SIRT1/Beclin-1/autophagy axis [48].

### 3.2 EC senescence induced by telomere shortening

Telomeres are located at the ends of chromosomes and consist of tandem repeat sequences of TTAGGG, which is rich in guanine and protects chromosomes from degradation, recombination, and fusion. Telomere length is regulated by telomeric reverse transcriptase (TERT), telomerase RNA component (TERC); and shelterin, a protein complex that protects chromosome ends [49]. In senescent cells, this repeat sequence is shortened during each cell division. When telomeres become very short, they lose the ability to maintain the T-loop structure that covers the chromosome ends. In 1995, Chang et al. demonstrated that in vascular ECs, telomere length gradually shortens with the occurrence of senescence [50]. Xu et al. revealed that ECs treated with homocysteine promoted EC senescence and telomere shortening with oxidative stress [51], and this was confirmed by Furumoto et al., who reported that intracellular vitamin C enrichment mitigated senescence-induced telomere shortening by inhibiting oxidative stress [52]. In addition, Shen et al. reported that IL-8 protects senescent HUVECs by promoting telomerase activity and upregulating TERT expression [53]. Telomere shortening has become a commonly used indicator to detect senescence in EC.

The key to cellular senescence lies in telomeres, the length of which determines the length of cellular life in some ways. The length of telomeres depends mainly on telomerase activity. TA-65 is a root extract of *Astragalus membranaceus* that substantially reduces vascular and brain senescence [54]. A study found that TA-65 increased telomerase activity in IMR90 cells [55]. When this extract was administered to senescent mice, their telomere length increased and their healthy lifespan was prolonged [56]; additionally, TA-65 was found to extend telomere length in subjects without adverse events in clinical studies [57, 58]. These studies suggest that maintaining telomere length by increasing telomerase activity is a highly feasible anti-senescence pathway.

### 3.3 EC senescence induced by epigenetic regulation

Epigenetics affects senescence through abnormal gene expression, transcription factor reactivation, genomic instability, and chromatin structural rearrangement [59]. Epigenetic factors regulate changes in cellular functions and thus affect EC senescence.

DNA methylation is an epigenetic mechanism that is regulated by a combination of methyltransferases and demethylases, and a central role for DNA methylation in vascular senescence-related diseases has been identified [60]. Ramini et al. analyzed genome-wide DNA methylation in normal and senescent HUVECs and noted a significant increase in demethylated sequences in senescent cells [61]. Zhang et al. reported that homocysteine promotes EC senescence by promoting hTERT promoter demethylation and downregulating hTERT expression in mouse aortic ECs [62]. Yang et al. revealed that the histone methyltransferase smyd3 increases the expression of H3K4me3 by binding to p21 and results in a senescent phenotype for vascular ECs and Ang II-induced senescence in mice [63].

In recent years, increasing evidence has indicated that acetylation modifications are involved in EC senescence. Chen et al. discovered that acetic acid promotes VEC senescence by enhancing the interaction between the SESAME complex and the acetyltransferase complex SAS, specifically promoting the acetylation of histone H4K16 in the region near telomeres and disrupting the telomeric heterochromatin structure to accelerate vascular EC senescence [64]. Lin et al. performed an overall lysine acetylome analysis in senescent HUVECs and found that 40 proteins associated with fatty acid metabolism had significantly reduced acetylation levels, thus also suggesting a relevance of EC senescence to acetylation [65]. SIRT is an NAD^+^-dependent deacetylase [66]. When DNA is damaged, SIRT1 and SIRT6 localize to the nucleus to promote cellular repair and anti-stress responses by mediating the tumor suppressor and DNA damage repair pathways. Inhibition of SIRT1 in ECs increases p53 activity [67], while SIRT6-induced senescence appears to act upstream of p53-p21 activation and SIRT6 as a key component in telomere protection and DNA repair [68]. SIRT3 is localized in mitochondria and protects ECs from oxidative stress [66] and senescence [69]. Furthermore, in senescent ECs, the expression of SIRT1, 6, and 3 is downregulated, which in turn causes secretion of endothelial SASP components by acting through the p53/p21 signaling pathway and exacerbates cellular senescence [66].

In addition, sirtuins are excellent targets for treatments to reduce endothelial senescence and may be used as small molecule activators to exert specific positive anti-senescence effects. Ginsenoside Rb1 reduced H_2_O_2_-induced HUVECs senescence by stimulating the SIRT1/AMP-activated protein kinase pathway [47]. Epimedium increases SIRT6 expression in aged mice and inhibits NF-κB protein expression and the inflammatory response in the body; these effects have the potential to delay senescence and extend lifespan [70, 71]. Currently, SIRT is the most critical anti-senescence target, and many anti-senescence studies have been conducted on SIRT-related activators, with herbal monomers accounting for more than half of the small-molecule activators. Therefore, SIRT1, SIRT6, and SIRT3 may be feasible anti-senescence targets for future research.

### 3.4 EC senescence induced by miRNA

A micro RNA (miRNA) is a small noncoding RNA (18-25 nucleotides) that regulates the expression of multiple genes at the post-transcriptional level, and in recent years, many researchers have investigated whether miRNA may be associated with EC senescence [72]. Hofmann et al. revealed that lncRNAH19 deletion in HUVECs resulted in the upregulation of p16 and p21 and thus accelerated ECs senescence; subsequently that team obtained consistent conclusions in a mouse model of EC-specific lncRNA H19 deficiency [73]. Moreover, miR-200c, miR-200a, and miR-200b are indispensable for the oxidative-stress-induced apoptosis and senescence in HUVECs, and miR-200c inhibits proliferation and induces senescence of HUVECs [74]. Menghini et al. found that miR-217 induced a senescence-like phenotype in HUVECs, HAECs and HCAECs by inhibiting SIRT1 and endothelial-type nitric oxide synthase acetylation [75]. Zhang et al. demonstrated that miR-21-5p/203a-3p promoted HUVECs senescence by downregulating the mitochondrial fission protein Drp1 along with mitochondrial dysfunction and activation of the AMPK-p53/p16 pathway [76].

Many studies have demonstrated that the removal of senescent cells through modulation of miRNAs leads to delayed senescence. Kallistatin is an endogenous protein that alleviates vascular senescence by inhibiting the synthesis of miR-34a [77]. SGLT2 inhibitors, which include dapagliflozin, canagliflozin, ertugliflozin, and empagliflozin, are a series of novel oral hypoglycemic agents with antidiabetic properties [78]. Among these, dapagliflozin improves vascular function and delays vascular senescence in diabetic patients by acting on miRNAs, particularly by upregulating the expression of miR30e-5p and decreasing the expression of miR199a-3p[79]. Although the mechanism of action of miRNA interference in cellular senescence is not yet clear, targeted inhibition of senescence-related miRNA expression has emerged as a potential key strategy to inhibit senescence, and more clinical trials are needed to verify the safety and efficacy of this therapy.

Cellular senescence is the result of the “survival of the fittest”, and serves as a protective mechanism by which an organism responds to environments that are not conducive to cell growth. Different environmental stimuli have varying consequences. Under certain conditions, ECs undergo senescence. Stimulating factors in the cellular microenvironment that act on cells may cause DNA damage or changes in chromatin modifications, such as DNA methylation and non-coding RNA modifications. The above factors initiate the EC senescence and induce changes in a variety of key factors that maintain normal cellular life biological activity, such as p16 and p21, thus resulting in corresponding changes in molecular, morphological, metabolic, and functional phenotypes. The initiation of senescence protects the organism, either to prevent the accumulation of mutations causing cell carcinogenesis or to prevent the induction of errors caused by changes in the function of damaged cells. However, the normal physiological functions of ECs, such as secretion, barrier function, immune function, neovascularization, and material transport, are impaired to varying degrees after damage or alteration of the related DNA or chromatin, which is closely related to a variety of diseases, especially central neurological disorders such as stroke, atherosclerosis, vascular dementia, and Alzheimer's disease (AD).

## 4. Senescence of ECs and neurological disorders

Cerebral circulation plays an important role in maintaining CNS homeostasis. The cerebral circulatory system, consisting of arteries, small arteries, and capillaries, delivers oxygenated blood and nutrients to the brain. Vascular cells such as endothelial cells are components of the neurovascular unit (NVU), an essential functional and anatomical structure. ECs are closely joined to each other, enabling an efficient system for cerebral blood flow (CBF), maintenance of neuronal metabolic activity and an effective BBB[80]. Dysfunction of the EC and BBB can lead to a variety of neurological diseases, including stroke, AD, hypertension, and vascular dementia. Using in vitro BBB models, Yamazaki et al. indicated that the accumulation of senescent BMVECs impairs barrier integrity and alters tight junction structure [81]. Some researchers have found that the transport function of the BBB is significantly altered by increased EC senescence. A structurally healthy BBB transports circulating proteins through receptor-mediated transport. However, with age, this transport shifts to ligand-nonspecific vacuolar transport, which is inseparably linked to EC senescence [82]. Alterations in the transcriptional program of brain ECs mediate differential plasma uptake. Experimentally, normal ECs take up large amounts of RMT-transported transferrin and leptin, whereas senescent ECs shift to increased uptake of horseradish peroxidase, vesicle-transported immuno-globulins, and albumin [83].

In this chapter, we discuss the relevance of stroke, AD, hypertension and vascular dementia to EC senescence, with the hope of shedding light on the pathogenesis of thesis diseases and the options for their treatment.

### 4.1 EC senescence is a potential risk factor for stroke

Senescence of ECs causes vascular dysfunction, promotes atherosclerosis, and contributes to the development of age-related vascular diseases. Cerebral EC dysfunction is a characteristic feature of many neurological conditions, including ischemic and hemorrhagic stroke [84]. SIRT6 in ECs attenuates stroke and neurological deficits by maintaining BBB integrity [85]. When ECs undergo senescence, SIRT6 expression is reduced [68], which greatly increases the risk of stroke. Numerous studies have found that cerebral ischemia/reperfusion (I/R) injury is accompanied by significant production of ROS [86-88], which further intensifies ECs senescence and increases the severity of stroke, forming a positive feedback loop [33]. Qing et al. found that the expression of IL-6, IL-17 and other senescence markers was elevated in the peripheral blood of elderly patients with acute cerebral infarction and showed that IL-17 promotes ECs senescence subsequently contributing to the occurrence of acute cerebral infarction in humans [89]. Nagy et al. found increased release of endothelin-1, matrix metalloproteinase-9, and other senescence-related molecules from BMVECs in a model of cerebral ischemia [90]. Likewise, Cheon et al. found significantly elevated expression of endothelial MMP2 and MMP9 in a mouse model of ischemic stroke, and these two molecules are often considered to be key molecules in senescence-associated secretory phenotypes [91]. Recent studies have found that endothelial progenitor cells have great prognostic value in ischemic stroke because they provide new ECs to supplement the function of senescent ECs after a stroke [92]. Although the clinical application of endothelial progenitor cells is limited by their availability and premature senescence, their ability to restore EC function after damage suggests that they can serve as a basis from which to develop an effective treatment strategy for stroke. In the future, perhaps we reverse the senescent phenotype of ECs can be reversed to achieve an excellent prognosis for stroke patients.

### 4.2 EC senescence is a predisposing factor for atherosclerosis

Atherosclerosis, a chronic inflammatory disease of the blood vessels, is one of the major causes of cardiovascular disease in elderly people [93]. The presence of numerous senescent cells in the vasculature is closely associated with pathophysiological changes in atherosclerosis, and SASP secreted by senescent cells contribute to the progression and imbalance of atherosclerotic plaques [94]. Furthermore, Bürrig et al. demonstrated the presence of senescent ECs in human atherosclerotic plaques [95]. In a mouse model of atherosclerosis, Merat et al. found that elevated expression of adhesion molecule-1 in senescent aortic vascular cells leads to the development of oxidative stress and thus accelerates the atherosclerotic process [96]. Senescent ECs display several factors, including IL-1α, TNF-α, MMP-3, MMP-12, MMP-13, MCP-1, and VCAM-1, all of which are associated with atherosclerotic plaque formation [97], which is essential for atherogenesis. Jia et al. revealed that significant TNF-α-mediated downregulation of VCAM1 and IL-1β in mouse ECs reduced turbulent blood flow and the progression of atherosclerotic lesions in atherosclerotic mice, and they validated this finding in human umbilical vein ECs and human aortic ECs [98],Similarly, Yang et al. found that TNF-α-induced MMP -9 and intercellular adhesion molecule-1 (ICAM-1) expression contribute to atherosclerosis [99]. Senescent HVECs increase MCP-1 expression and subsequently recruit circulating monocytes, which differentiate into macrophages in the submucosa and are involved in cholesterol metabolism in atherosclerosis [100]. Barton et al. identified a significant increase in the total density of endothelin-1 binding in the vessel wall and atheromatous plaques due to an increased density of endothelin receptors [101]. Additional investigations have indicated that hyperphosphatemia induces human EC senescence by increasing endothelin-1 production and that HVEC senescence promotes endothelin-1 expression [102]. Nevertheless, increased endothelin-1 inhibits the release of NO from ECs, which impairs endothelium-dependent relaxation and promotes atherosclerosis formation [100]. In senescent ECs, SASPs increase the activity of CCAAT enhancer binding protein β and NF-κB in an autocrine feedforward loop and amplifies SASP signaling [24, 25], thus altering the phenotype of non-senescent neighboring cells through paracrine effects and thereby promoting atherosclerosis [26].

### 4.3 EC senescence interacts with hypertension

Hypertension is the largest contributor to morbidity and mortality worldwide [103], but few studies have shown a relationship between EC senescence and hypertension. However, the expression of vasoprotective and vasoconstrictor molecules is altered during EC senescence, which is an important cause of increased vascular resistance and hypertension [104]. In addition, the rate of telomere loss is significantly increased in ECs of patients with hypertension [105]. Hypertension drives EC senescence, further decreasing nitric oxide expression and increasing levels of endothelin 1 and angiotensin II [106, 107], which brings about further increases in blood pressure and more severe senescence through a feedback loop. Nguyen et al. revealed a significant increase in phosphorylation of endothelial NO synthase residue threonine 495 (eNOSThr495) by IL-17 in cellular and mouse models and thus an increase in systolic blood pressure [108], accompanied by Li et al.'s identification of IL-17 as critical for EC senescence [109], implying that IL-17 is a mediator of the essential link between senescence and hypertension in ECs. Endocan, encoded by the endothelial cell-specific molecule-1 (ESM-1) gene, is a proteoglycan, and Klisic et al. found that endocan is closely associated with adult hypertensive populations [110], while key molecules that regulate endocan expression, such as TNF-α and IL-1β [111] are associated with EC senescence.

### 4.4 EC senescence is a potential pathogenetic factor for Alzheimer's disease

AD is a neurodegenerative disorder characterized by progressive memory and cognitive decline [112]. Although the mechanisms underlying age-related AD susceptibility are unknown, the effects have been well studied [113]. Senescent ECs can be detected in the frontal and temporal cortices of patients with AD[114], and the expression of the cellular senescence marker PAI-1 is significantly elevated [115]. This suggests that the senescence of ECs plays a crucial role in the development of AD. In a cellular model of AD using HMVECs, Dias et al. demonstrated a significant elevation of IL-6 secreted by cells [116], and exposure of brain ECs to Aβ1-40 induced elevated expression of MCP-1, IL-1β, and IL-6 [117]. Liu et al. analyzed rat brain microvascular ECs and found that IL-1β, IL-6 and TNF-α upregulated the expression of ICAM-1 and VCAM-1 [118]. Tripathy et al. demonstrated a significant increase in HIF-1α, IL-6, MCP-1, MMPs and ROS in AD mouse models as well as in cultured brain ECs [119]. Based on the above experiments, multiple EC senescence markers were detected, suggesting that they may play a role in AD. Furthermore, amyloid β (Aβ) deposition in the brain is a major pathological feature of AD and is thought to be a driving factor in the progression of AD[120]. An imbalance between Aβ production and clearance, especially due to impaired Aβ clearance, drives Aβ deposition and eventual plaque formation in the brains of AD patients [121]. Vascular ECs, an important component of the BBB, are thought to be involved in Aβ transport and clearance [122, 123]. During the process of Aβ clearance, Aβ passes through the cytosolic membrane of cerebrovascular ECs (brain tissue side) into the cytoplasm and across the luminal side cytosol to the peripheral blood, a process through which some Aβ may be cleared directly by the EC clearance system [124, 125]. The transport proteins low-density lipoprotein receptor-related protein 1(LRP1), receptor for advanced glycosylation end products, major histocompatibility complex class I-related protein, P-glycoprotein (P-gp), ABCA1, BCRP, and ABCC1 may be involved in the transport and clearance of Aβ across cerebrovascular ECs [124]. Osgood et al. found that LRP1 expression on the surface of ECs was significantly lower in 34-month-old rats than in 3-month-old rats [126]. Similarly, Silverberg et al. identified a decrease in the expression of LRP1 and P-gp by ECs with increasing EC senescence in a senescent rat model [127], which in turn led to impaired clearance transport mechanisms for Aβ and massive deposition of Aβ in the brain, facilitating the progressive development of AD. Many studies have demonstrated that the toxic effects of Aβ cause dysregulation of receptor and transporter expression in ECs [128]. Excess Aβ induces chronic inflammation by promoting oxidative stress, impairing the structure and function of ECs, and inducing the onset of senescence [129], which further aggravates the progression of AD. Targeting senescent ECs is a promising direction in AD research and is likely to provide new clinical treatment strategies for Aβ clearance in the brains of patients with AD.

### 4.5 Vascular dementia is a possible consequence of EC senescence

Vascular dementia (VaD) is a common cognitive disorder responsible for at least 20% of dementia cases, which makes it second only to AD in prevalence. VaD has a much earlier the age of onset than AD; patients as young as 20 years have been encountered in clinical practice [130]. VaD is usually triggered by cerebrovascular disease, in which cerebrovascular ECs are vulnerable. Low NO production in senescent ECs [131] contributes to the development of vascular dementia through the onset of excessive vasoconstriction, disturbances in cerebral blood flow regulation, and weakening of neuroprotection [132]. De la Torre et al. noted reduced mitochondrial content and loss of TJs in brain ECs of patients with VaD [133], indicating that EC senescence alters the structure of the BBB [129], a key part of the pathological alterations in VaD. The increased expression of the bone-development proteins MMP-13 and CD36 after BBB damage further contributes to the development of VaD and cause a correlation between VaD and AD [134].

## 5. Discussion and Conclusion

Senescence is initiated when cells are damaged, and senescent cells are then removed by immune cells. When senescent cells are not removed in a timely manner and instead accumulate gradually, they eventually contribute to various diseases.

In this review, we explored the phenotypes and molecular mechanisms of EC senescence., We discussed the associations between EC senescence and several neurological disorders. As the principal and most complex organ of the human body, the brain requires a stable, fine-tuned internal environment to function properly, and the BBB acts as a "gatekeeper" in this regard. Both the structural and the functional integrity of the BBB are closely related to the course of numerous neurological disorders, and dysregulation of the BBB can result in serious neurological disorders. ECs act as a significant component of the BBB, and senescence is the predetermined fate of all cell types, including ECs, whether physiologically or pathologically. Research on EC senescence is extremely valuable. Atherosclerosis, hypertension, AD, and other neurological disorders are associated with EC dysfunction and senescence. Multiple symptoms co-occur with neurological disorders. Patients with hypertension havean increase probability of developing complications such as stroke or atherosclerosis at a later stage, and the exploration of the mechanisms of various neurological disorders may go in the same direction and eventually point to the senescence of ECs. Of course, the above is only a reasonable hypothesis, and more research is needed. It has been demonstrated that senescent ECs have potential as new therapeutic targets and can provide new therapeutic strategies for cardiovascular treatment and prevention.

Owing to the unique limitations of this research area, many issues remain to be addressed. The first is the construction of relevant models, which is certainly made more difficult by the fact that the function of ECs is dependent on the interaction of many other cell types, such as smooth muscle cells, making it difficult to exclude the influence of other factors in many animal model studies. This is now potentially attainable with advances in technology, and through histological approaches such as single-cell sequencing, we can further understand the unique expression profile of senescent ECs and further clarify the underlying mechanisms of senescence. In addition, understanding how ECs senesce not only helps to specifically eliminate senescent cells but also facilitates targeted therapy of certain neurological disorders.
